# The impact of smartphone dependence on college students’ sleep quality: the chain-mediated role of negative emotions and health-promoting behaviors

**DOI:** 10.3389/fpubh.2024.1454217

**Published:** 2024-09-19

**Authors:** Yunfei Tao, Zhaozhi Liu, Li Huang, Haowei Liu, Haodong Tian, Jinlong Wu, Lan Li, Li Peng

**Affiliations:** College of Physical Education, Southwest University, Chongqing, China

**Keywords:** college students, health-promoting behaviors, negative emotions, sleep quality, smartphone dependence

## Abstract

**Objective:**

Sleep disturbances among college students have become a significant issue affecting their daily lives. This study aims to explore the relationship between smartphone dependence and sleep quality and examine the mediating roles of negative emotions and health-promoting behaviors.

**Methods:**

A total of 23,652 college students were included in the study, and 21,314 valid questionnaires were collected. The survey assessed demographic factors, smartphone dependence, sleep quality, negative emotions, and health-promoting behaviors. A chain mediation analysis was conducted to examine the relationships among these factors.

**Results:**

Smartphone dependence was significantly positively correlated with sleep quality (*r* = 0.272, *p* < 0.001) and negative emotions (*r* = 0.414, *p* < 0.001), and significantly negatively correlated with health-promoting behaviors (*r* = −0.178, *p* < 0.001). Sleep quality was positively correlated with negative emotions (*r* = 0.472, *p* < 0.001) and negatively correlated with health-promoting behaviors (*r* = −0.218, *p* < 0.001).Smartphone dependence was a significant positive predictor of sleep quality. Moreover, negative emotions and health-promoting behaviors influenced the relationship between smartphone dependence and sleep quality. The total effect, direct effect, and indirect effect values were 0.304, 0.122, and 0.170, respectively.

**Conclusion:**

Different demographic factors (such as gender and place of residence) can lead to variations in different variables. Smartphone dependence and negative emotions have a positive impact on sleep quality among college students, while health-promoting behaviors have a negative impact. Smartphone dependence directly and positively affects sleep quality and can also influence it indirectly through the mediating effects of negative emotions and health-promoting behaviors, both individually and in a chain-like manner.

## 1 Introduction

With the ongoing advancements in mobile technology, people’s modes of communication, learning, work, and entertainment have been significantly enriched, making smartphones an indispensable part of daily life. According to recent data, as of October 2023, China had 1.092 billion internet users, 99.9% of whom accessed the internet via smartphones. The number of online video users reached 1.067 billion, marking an increase of 36.13 million since December 2022, with the trend continuing to rise (1). College students, a primary demographic of smartphone users, are particularly prone to developing smartphone dependence. The “2024 China Resident Sleep Health White Paper” reveals that 56% of college students spend over 8 h per day on their phones (2). This excessive use is largely attributed to the transition from adolescence to adulthood and the lack of smartphone restrictions in college, following the stringent controls experienced during high school, which can foster excessive dependence (3). Furthermore, due to the demanding coursework and extracurricular activities during the day, college students are more likely to use their phones before bedtime as a means of finding personal space and emotional comfort. However, this practice often results in reduced sleep duration and exacerbates sleep dependency (4).

Smartphone dependence is increasingly recognized as a behavioral addiction, characterized by cravings, avoidance, and a reduction in health-promoting behaviors (5). Excessive smartphone use can lead to dependency, with studies showing that some college students struggle with controlling their usage. This addiction is significantly correlated with negative emotions such as depression (6) and poor sleep quality (4), indicating that increased smartphone dependence can elevate psychological health risks and reduce sleep quality. Moreover, the light and electromagnetic fields emitted by smartphones can alter brain activity, stimulating emotional and cognitive functions and heightening arousal, which further contributes to sleep disorders (7). Additionally, smartphone dependence can diminish health-promoting behaviors, exacerbating the accumulation of negative emotions. For example, dependence reduces social support capabilities, leading to less interpersonal communication and increased negative emotions (8). Conversely, reducing smartphone use and increasing physical activity can effectively improve mental health and sleep quality (9). Thus, smartphone dependence can negatively impact sleep quality by reducing health-promoting behaviors and/or increasing negative emotions. The relationship between smartphone dependence and sleep quality in college students appears to be dynamic. Research indicates a complex bidirectional predictive relationship between smartphone dependence and sleep quality, influenced by various factors, and a similar bidirectional relationship exists between sleep quality and negative emotions like depression (10). This suggests the need for longitudinal studies to further explore the relationship between smartphone addiction and sleep quality beyond cross-sectional findings.

Negative emotions such as anxiety, depression, and stress are key indicators of mental health among college students. Studies have shown a bidirectional relationship between smartphone dependence and negative emotions, where each can positively predict the other, and both contribute to poor sleep quality and exacerbated sleep disorders (11). College students often experience accumulated negative emotions due to lifestyle changes, academic pressures, and career challenges (12, 13). The unrestricted leisure time in college, particularly for those without strong hobbies, can lead to excessive smartphone use, which further isolates them socially, reduces health-promoting behaviors like outdoor activities, and decreases sleep quality due to prolonged screen time and heightened brain activity before bed (6, 14). Given the reciprocal relationship between smartphone dependence and negative emotions, addressing negative emotions has been found to be effective in mitigating smartphone use issues and improving sleep disorders (15). Therefore, there may be a more complex relationship between negative emotions, smartphone dependence, and sleep quality that warrants further exploration.

Health-promoting behaviors refer to the lifestyle choices individuals make to enhance their health, wellbeing, and self-actualization. These behaviors are “multidimensional, spontaneous, and continuous” daily activities, including nutrition and exercise (16). Research shows a significant negative predictive relationship between health-promoting behaviors and negative emotions among college students, where engaging in these behaviors can alleviate negative emotions, while accumulated negative emotions can reduce the level of health-promoting behaviors (17). Additionally, health-promoting behaviors are crucial for sleep quality, as unhealthy lifestyle choices can adversely affect sleep. Most studies have focused on harmful behaviors like sedentary activity, drinking, and smoking (18, 19), with fewer exploring the impact of smartphone dependence on health-promoting behaviors and their influence on sleep quality from a holistic perspective (20). This connection highlights the potential of health-promoting behaviors to mitigate smartphone dependence and improve overall wellbeing, suggesting a link between these factors.

Based on this, negative emotions and health-promoting behaviors can serve as mediating factors in the relationship between smartphone dependence and sleep quality among college students. They may also jointly act as chain mediators influencing this relationship. Most current research on smartphone dependence and sleep quality in college students focuses on isolated emotions such as anxiety or depression, with only a few studies incorporating overall negative emotions or health-promoting behaviors, either independently or in combination, to examine their impact on smartphone dependence or sleep quality. Therefore, the results of this study aim to understand the role of smartphone dependence in improving sleep quality through its influence on negative emotions and health-promoting behaviors. Additionally, it will help explore the effectiveness of health-promoting behaviors in mitigating smartphone dependence and sleep disorders. This research provides important evidence and theoretical support for preventing and intervening in smartphone dependence among college students, thereby maintaining their physical and mental health and improving sleep quality.

## 2 Materials and methods

### 2.1 Participants

**Figure 1:**
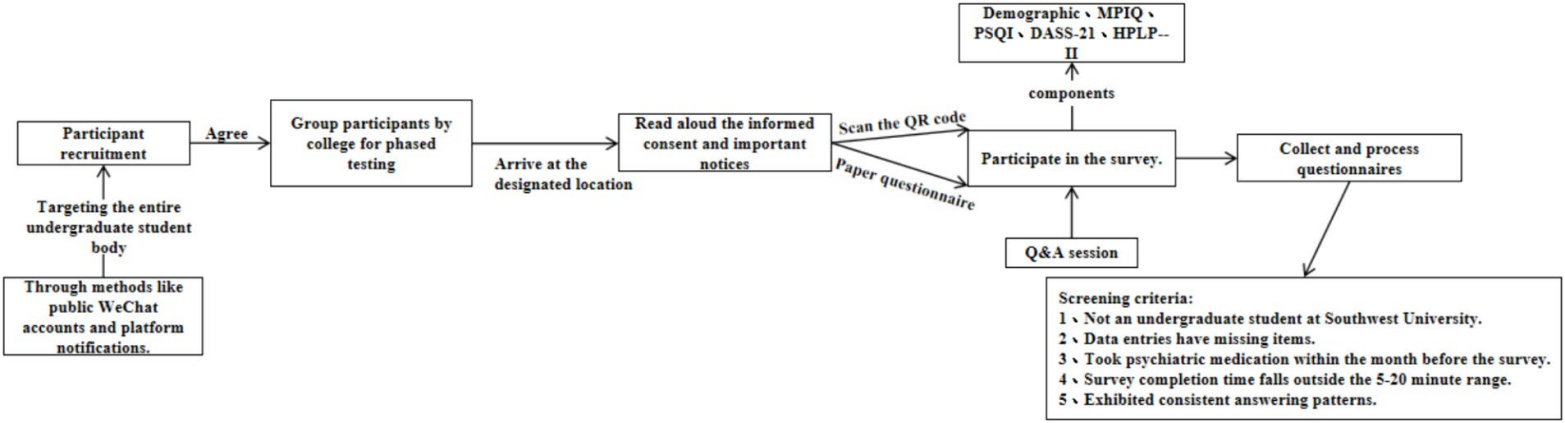
Research design and survey process flowchart.

This cross-sectional study was conducted from October to December 2021, targeting all undergraduate students at Southwest University, excluding senior students (fourth-year students). A simple random sampling method was employed to conduct the survey, inviting all undergraduate students, except seniors, to participate online. The questionnaire consisted of five parts: demographic information (e.g., gender, place of residence, parents’ education level, etc.), the Health-Promoting Lifestyle Profile II (HPLP-II), the Depression Anxiety Stress Scales (DASS-21), the Mobile Phone Involvement Questionnaire (MPIQ), and the Pittsburgh Sleep Quality Index (PSQI). During the survey, students were organized by their respective colleges to gather at designated locations, where data collection was carried out using both mobile phone scanning and paper scales. Trained staff, who had received standardized training, read aloud the informed consent and survey instructions. Each questionnaire included an informed consent section, which participants needed to agree to and sign themselves. The detailed research process is illustrated in Figure 1.

A total of 29,754 students were invited to participate in this survey, with 23,652 students agreeing to participate. Among them, 21,314 valid questionnaires were collected, resulting in a response rate of 79.49% and a valid response rate of 90.12%. The sample included 10,338 male students (48.50%) and 10,976 female students (51.5%).Inclusion criteria were as follows: (1) current undergraduate students at Southwest University; (2) no missing items in the questionnaire responses; (3) no psychiatric medications taken within 1 month prior to data collection; (4) survey completion time between 5 and 20 min, as specified by the questionnaire design; (5) absence of response patterns indicating regularity or inconsistency. Based on the G-POWER sample size calculation formula, the required minimum sample size was determined to be 384, using the formula *n* = Z^2 × p × (1 − p)/E^2, where Z = 1.96 (with a 95% confidence level), *p* = 0.5, and E (margin of error) = 0.05. This study met the minimum sample size requirement. The survey was conducted with the consent of college leaders, teachers, and the participants themselves, ensuring voluntary participation and data confidentiality. In accordance with the Declaration of Helsinki, the study was approved by the Ethics Review Committee of the Southwest University Hospital (Approval no: SWH202011281421).

### 2.2 Research tools

#### 2.2.1 Smartphone dependence

The Mobile Phone Involvement Questionnaire (MPIQ), modified by Walsh et al. (21), and translated into multiple languages for global use, assesses individuals’ dependence on smartphone use, including related behaviors and psychological traits. The MPIQ consists of eight items, each rated on a 7-point Likert scale, with scores ranging from 8 to 56. Higher scores indicate stronger dependence on smartphone use (22). The MPIQ demonstrates good reliability and validity, with an internal consistency coefficient (Cronbach’s *α*) of 0.84 (23). In this study, the Cronbach’s *α* value for the MPIQ was 0.902.

#### 2.2.2 Pittsburgh sleep quality index

The Pittsburgh Sleep Quality Index (PSQI), developed and revised by Buysse et al. (24), is designed to assess individual sleep quality and disturbances. This scale has been translated into multiple languages, including Chinese, and is widely used globally. The Chinese version of the PSQI consists of seven subcomponents: “subjective sleep quality,” “sleep latency,” “sleep duration,” “sleep efficiency,” “sleep disturbances,” “use of sleeping medication,” and “daytime dysfunction.” Each item is scored from 0 to 3, and the total PSQI score is the sum of the scores of these seven subcomponents, ranging from 0 to 21. Higher scores indicate more severe sleep disturbances. Specifically, a total score of 0–5 indicates “good” sleep quality, 6–10 indicates “mild” sleep disturbances, 11–15 indicates “moderate” sleep disturbances, and 16–21 indicates “severe” sleep disturbances. The PSQI demonstrates good reliability and validity, with an internal consistency coefficient (Cronbach’s *α*) of 0.83 (25). In this study, the Cronbach’s *α* value for the PSQI was 0.87.

#### 2.2.3 Negative emotion

The Depression Anxiety Stress Scales (DASS-21), developed by Lovibond and Lovibond (26), has been translated into multiple languages, including Chinese. This scale is widely used in China and has been proven effective in measuring levels of depression, anxiety, and stress within negative emotional states. The Chinese version of the DASS-21 demonstrates good reliability and validity, with internal consistency coefficients (Cronbach’s *α*) for the depression, anxiety, and stress subscales being 0.83, 0.80, and 0.82, respectively. The overall DASS score has a Cronbach’s *α* of 0.92 (27). In this study, the Cronbach’s *α* value for the DASS-21 was 0.957. The scale comprises three dimensions: anxiety, depression, and stress, with each dimension containing seven items, totaling 21 items. Responses are scored on a four-point scale: “0” for “never,” “1” for “sometimes,” “2” for “often,” and “3” for “almost always.” Higher scores on the scale indicate more severe negative emotions.

#### 2.2.4 Health-promoting behaviors

The Health-Promoting Lifestyle Profile II (HPLP-II) scale, developed by Walker et al. (28), based on Pender’s Health Promotion Model, is designed to effectively measure health-promoting behaviors and their various dimensions. The Chinese version of the HPLP-II demonstrates good reliability and validity and is widely used to assess the lifestyles of university students. This scale is a well-established tool for assessing health behaviors both domestically and internationally. It includes 52 items divided into six subscales: self-actualization (nine items), health responsibility (nine items), physical activity (eight items), nutrition (nine items), interpersonal relations (nine items), and stress management (eight items). The questionnaire has good reliability and validity, with Cronbach’s *α* coefficients as follows: self-actualization (0.904), health responsibility (0.814), physical activity (0.809), nutrition (0.757), interpersonal relations support (0.800), stress management (0.702), and the overall Health-Promoting Lifestyle Profile (HPLP) (0.922). In this study, the Cronbach’s *α* value for the HPLP-II was 0.981. The scale uses a four-point Likert scoring method (1 = never, 2 = sometimes, 3 = often, 4 = routinely), with scores ranging from 52 to 208. The scoring is as follows: 52–90 points indicate “poor,” 91–129 points indicate “average,” 130–168 points indicate “good,” and 169–208 points indicate “excellent.” Higher scores represent higher levels of health-promoting behaviors.

### 2.3 Statistical analyses

This study conducted statistical analysis using IBM SPSS version 27.0. First, descriptive statistics were performed, with frequencies and percentages used to describe the demographic characteristics of the participants. Cronbach’s alpha was used to verify the internal consistency coefficients and reliability of the four scales. Pearson correlation analysis was conducted on the independent variable, dependent variable, and the two mediating variables. Harman’s single-factor test was employed to assess common method bias across all variables. For the chain mediation analysis, the SPSS PROCESS macro v4.1 developed by Hayes was used. Specifically, Model 6 in PROCESS was utilized for mediation effect analysis, with 5,000 Bootstrap resamples and bias-corrected percentile Bootstrap confidence intervals (CIs) used to evaluate effect sizes. A mediation effect was considered significant when the 95% CI did not include zero. Additionally, during the analysis, covariates such as gender, place of household registration, and parents’ education levels were included in the model to control for demographic factors while exploring the chain mediation effect of health-promoting behaviors on negative emotions. Finally, linear regression analysis was conducted, with the MPIQ, PSQI, DASS-21, and HPLP-II used as outcome variables, while other demographic factors and variables were treated as predictors to explore their predictive relationships with the outcome variables.

## 3 Results

### 3.1 Common method bias test

To avoid common method bias, procedural controls were implemented to mitigate its sources. An exploratory factor analysis of all test items was conducted using Harman’s single-factor test in SPSS version 25.0. The results indicated that the first factor accounted for 35.386% of the variance, which is below the critical threshold of 40%, suggesting that there is no significant common method bias in this study.

### 3.2 Descriptive analysis

Before examining the mediating effects of negative emotions and health-promoting behaviors, descriptive statistics were performed on the collected demographic variables, including gender, place of residence, and parents’ educational levels (Table 1).

**Table 1:** Demographic characteristics.

Variable	Level	*N*	Percentage (%)
Gender	Male	10,338	48.50
	Female	10,976	51.50
Place of residence	Rural	10,775	50.55
	Urban	10,539	49.45
Father’s education	Primary or below	2,486	11.66
	Middle school	6,747	31.66
	Associate degree	6,815	31.97
	Bachelor’s or above	5,266	24.71
Mother’s education	Primary or below	4,020	18.86
	Middle school	6,450	30.26
	Associate degree	6,607	31.00
	Bachelor’s or above	4,237	19.88
Only child	Yes	7,827	36.72
	No	13,487	63.28
Smoking	Yes	753	3.53
	No	20,561	96.47
Alcohol consumption	Yes	454	2.13
	No	20,860	97.87

### 3.3 Correlation analysis

The correlation results (Table 2) indicate that college students who maintain higher levels of health-promoting behaviors report significantly lower levels of mobile phone dependency, sleep quality issues, and negative emotions. Conversely, higher levels of mobile phone dependency are associated with more severe sleep disturbances and negative emotions. The correlations among all variables were significant, meeting the prerequisites for conducting a chain mediation analysis.

**Table 2:** Correlation analysis between mobile phone dependence, sleep quality, negative emotions, and health-promoting behaviors.

Regression equation		Overall fit index			Significance		
Outcome variable	Predictor variable	R	R^2^	F	*β*	SE	*t*
PSQI	MPIQ	0.299	0.090	262.295***	0.298	0.007	42.619
	Gender				0.081	0.013	6.101
	Place of residence				0.013	0.016	0.812
	Father’s education				−0.003	0.010	−0.298
	Mother’s education				−0.022	0.010	−2.261
	Smoking				0.228	0.053	4.282
	Drinking				0.387	0.050	7.697
	Only child				−0.045	0.016	−2.833
PSQI	MPIQ	0.490	0.240	641.545***	0.118	0.007	16.994
	DASS-21				0.423	0.007	62.239
	HPLP-II				−0.083	0.006	−13.483
	Gender				0.083	0.012	6.816
	Place of residence				0.030	0.015	2.018
	Father’s education				0.000	0.009	−0.018
	Mother’s education				−0.013	0.009	−1.471
	Smoking				0.165	0.049	3.380
	Drinking				0.256	0.046	5.571
	Only child				−0.055	0.014	−3.818
HPLP-II	MPIQ	0.232	0.054	134.862***	−0.087	0.008	−11.219
	DASS-21				−0.083	0.008	−10.870
	Gender				0.240	0.014	17.731
	Place of residence				0.020	0.017	1.184
	Father’s education				−0.051	0.010	−5.081
	Mother’s education				−0.045	0.010	−4.609
	Smoking				−0.285	0.055	−5.222
	Drinking				−0.071	0.052	−1.375
	Only child				−0.151	0.016	−9.390
DASS-21	MPIQ	0.400	0.160	506.375***	0.397	0.006	62.108
	Gender				0.040	0.012	3.327
	Place of residence				−0.035	0.015	−2.348
	Father’s education				−0.016	0.002	−0.789
	Mother’s education				−0.029	0.009	−3.286
	Smoking				0.092	0.049	1.890
	Drinking				0.288	0.046	6.255
	Only child				−0.005	0.014	−0.326

***Indicates *p* < 0.001.

### 3.4 Hierarchical regression analysis of chain mediation effect

Based on hierarchical regression analysis, the following model was established: sleep quality was set as the dependent variable, with mobile phone dependence, negative emotions, and health-promoting behaviors as independent variables. Gender, place of residence, and parents’ educational levels were included as control variables to test the main, direct, and indirect effects (Table 3). The results showed that mobile phone dependence significantly positively predicted sleep quality and negative emotions (*β* = 0.298, *p* < 0.001; *β* = 0.397, *p* < 0.001) and significantly negatively predicted health-promoting behaviors (*β* = −0.087, *p* < 0.001). When both health-promoting behaviors and negative emotions were included in the regression equation, negative emotions significantly positively predicted sleep quality, and health-promoting behaviors significantly negatively predicted sleep quality (*β* = 0.423, *p* < 0.001; *β* = −0.083, *p* < 0.001). Additionally, mobile phone dependence significantly positively predicted sleep quality (*β* = 0.118, *p* < 0.001).

**Table 3:** Hierarchical regression analysis of chain mediation effect.

	M	SD	MPIQ	PSQI	DASS-21	HPLP-II
MPIQ	29.83	10.48	1			
PSQI	6.18	2.87	0.272***	1		
DASS-21	33.04	12.15	0.414***	0.472***	1	
HPLP-II	134.38	29.89	−0.178***	−0.218***	−0.264***	1

***Indicates *p* < 0.001.

### 3.5 Chain mediation effect analysis

**Figure 2:**
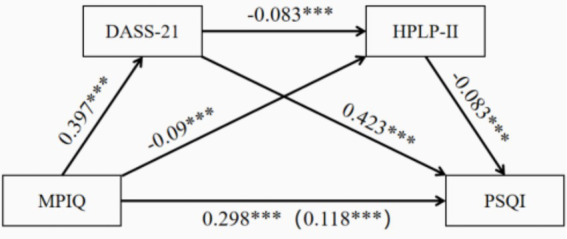
Mediation effects of negative emotions and health-promoting behaviors on the relationship between mobile phone dependence and sleep quality.

Emotions and health-promoting behaviors significantly mediated the relationship between mobile phone dependence and sleep quality (Table 4, Figure 1). The total mediation effect, with negative emotions and health-promoting behaviors as mediators, was 0.170, accounting for 56.06% of the total effect of mobile phone dependence on sleep quality (total effect value 0.304). The mediation effect included three indirect pathways: mobile phone dependence → negative emotions → sleep quality (effect value 0.163), mobile phone dependence → health-promoting behaviors → sleep quality (effect value 0.006), and mobile phone dependence → negative emotions → health-promoting behaviors → sleep quality (effect value 0.002). These indirect effects accounted for 53.49, 1.84, and 0.72% of the total effect, respectively, and the 95% confidence intervals (CIs) for these three indirect effects did not include zero, indicating that all three indirect effects were significant. Additionally, the comparison of mediation effects showed significant differences between the mediation effects of negative emotions and health-promoting behaviors (95% CI: 0.149, 0.165), and both the mediation effects of negative emotions and health-promoting behaviors were significantly different from the total chain mediation effect (95% CI: 0.152, 0. 168; 95% CI: 0.002, 0.005) (Figure 2).

**Table 4:** Analysis of direct effects of mobile phone dependence on sleep quality and mediation effects of negative emotions and health-promoting behaviors.

	Effects	Boot SE	Boot LLCI	Boot ULCI	Relative mediation effect
Aggregate effect	0.304	0.007	0.290	0.318	
Total direct effect	0.122	0.007	0.109	0.136	40.29%
Total indirect effect	0.170	0.004	0.162	0.178	56.06%
Indirect effect 1	0.163	0.004	0.155	0.171	53.49%
Indirect effect 2	0.006	0.001	0.004	0.007	1.84%
Indirect effect 3	0.002	0.001	0.002	0.003	0.72%
Compare 1	0.157	0.004	0.149	0.165	
Compare 2	0.160	0.004	0.152	0.168	
Compare 3	0.003	0.001	0.002	0.005	

### 3.6 Explicit regression analysis of variables in the chain mediation model

Four linear regression analyses were conducted, with each of the four variables in the model being used as the outcome variable in turn (as shown in Table 5). Demographic factors and the remaining variables were used as predictor variables. The results showed the following:

**Table 5:** Regression analysis of variables in the chain mediation model.

Outcome variable	Predictor variable	R	*R*^2^	F	*β*	*t*	*P*
MPIQ	Gender	0.077	0.006	18.079	0.039	5.709	<0.001
	Residence				0.029	3.449	<0.010
	Father’s education				−0.005	−0.490	0.624
	Mother’s education				0.011	1.130	0.259
	Only child				−0.024	−3.030	<0.010
	Smoking				−0.012	−1.531	0.126
	Drinking				0.057	7.567	<0.001
PSQI	Gender	0.110	0.012	37.117	0.051	7.461	<0.001
	Residence				0.015	1.747	0.081
	Father’s education				−0.004	−0.423	0.672
	Mother’s education				−0.019	−1.854	0.064
	Only child				−0.028	−3.569	<0.001
	Smoking				0.028	3.682	<0.001
	Drinking				0.072	9.519	<0.001
DASS-21	Gender	0.087	0.008	23.418	0.036	5.299	<0.001
	Residence				−0.007	−0.811	0.417
	Father’s education				−0.018	−1.838	0.066
	Mother’s education				−0.026	−2.581	<0.05
	Only child				−0.012	−1.486	0.137
	Smoking				0.009	1.140	0.254
	Drinking				0.066	8.727	<0.001
HPLP-II	Gender	0.190	0.036	113.971	0.113	16.688	<0.001
	Residence				0.008	0.953	0.341
	Father’s education				−0.047	−4.847	<0.001
	Mother’s education				−0.044	−4.455	<0.001
	Only child				−0.069	−8.935	<0.001
	Smoking				−0.038	−5.139	<0.001
	Drinking				−0.020	−2.691	<0.01

When MPIQ was the outcome variable, gender (*β* = 0.039, *p* < 0.001), residence (*β* = 0.029, *p* < 0.01), alcohol consumption (*β* = 0.057, *p* < 0.001) were positive predictors.

When PSQI was the outcome variable, gender (*β* = 0.051, *p* < 0.001), smoking status (*β* = 0.028, *p* < 0.001), alcohol consumption (*β* = 0.072, *p* < 0.001) were positive predictors.

When DASS-21 was the outcome variable, gender (*β* = 0.036, *p* < 0.001), alcohol consumption (*β* = 0.066, *p* < 0.001) were positive predictors.

When HPLP-II was the outcome variable, only gender (*β* = 0.113, *p* < 0.001) served as a positive predictor.

## 4 Discussion

This study examined the impact of smartphone dependence on sleep quality among college students, exploring the mediating roles of negative emotions and health-promoting behaviors. The results indicate a positive correlation between smartphone dependence and poor sleep quality. Health-promoting behaviors were found to be negatively correlated with smartphone dependence, sleep quality, and negative emotions. Moreover, smartphone dependence not only directly and positively affects sleep quality but also influences it through the independent and combined mediating effects of negative emotions and health-promoting behaviors.

The results indicate a positive correlation between smartphone dependence and poor sleep quality, meaning that the more severe the smartphone dependence among college students, the lower their sleep quality and the more serious their sleep disturbances. Similar to previous studies, smartphone dependence is identified as a significant factor affecting sleep quality (29). This relationship arises from increased academic pressure, reduced leisure time, and the fragmented nature of internet entertainment. Many students use their phones before sleep to relax, but this often leads to heightened anxiety and stress (30), inducing the brain to release more dopamine, which keeps the brain active, reduces sleep time, and causes sleep disturbances (31). Further demographic analysis reveals that female students, those living in rural areas, non-only children, and those with a history of alcohol consumption are more likely to develop higher smartphone dependence. Additionally, female students, non-only children, and those with smoking and drinking histories are more prone to experiencing severe sleep disturbances. This suggests that when studying smartphone dependence and sleep disorders among college students, it is essential to consider these demographic factors separately for a more nuanced understanding. It also highlights that certain groups, such as females, non-only children, and students with a history of alcohol consumption, may be at greater risk for severe smartphone dependence and sleep disorders.

The mediation effect of negative emotions reveals that smartphone dependence indirectly influences sleep quality through the independent mediation of negative emotions. Additionally, there is a significant positive relationship between smartphone dependence and negative emotions among college students. Similar to existing findings, negative emotions and smartphone dependence interact, where increased smartphone use exacerbates negative emotions, leading to more severe sleep disturbances. Furthermore, the accumulation of negative emotions may also increase smartphone dependence, as students may rely on their phones as a means of relaxation. However, this reliance does not alleviate negative emotions and instead results in reduced sleep time and heightened smartphone dependence (4). Moreover, studies suggest that accumulated negative emotions adversely affect sleep quality, such as emotional induction significantly delaying sleep onset latency (32). Anxiety and depression are identified as key factors contributing to poor sleep quality (33). Demographic analysis also indicates that female students, non-only children, and those who smoke or drink are more likely to experience severe sleep disturbances. These findings collectively suggest that negative emotions are both a direct factor affecting sleep quality and a mediating factor in the relationship between smartphone dependence and sleep quality.

Although health-promoting behavior was a statistically-significant mediator in the chain-mediation between mobile phone dependence and sleep quality, this effect was weak. The mediating effect of negative mood was much more important. To be specific, the mediation effect of health-promoting behaviors demonstrates that smartphone dependence indirectly influences sleep quality through this independent mediator. There is a significant negative correlation between smartphone dependence and health-promoting behaviors among college students, and both smartphone dependence and sleep quality are negatively correlated with health-promoting behaviors. Consistent with existing findings, more severe smartphone dependence leads to lower levels of health-promoting behaviors and more serious sleep disturbances (34, 35).Health-promoting behaviors include six dimensions: interpersonal relations, health responsibility, stress management, nutrition, physical activity, and spiritual growth. These dimensions can assess the daily lifestyle behaviors of most students. The study found that factors such as gender, alcohol consumption, and only-child status significantly impact the level of health-promoting behaviors. For example, males might have higher levels due to greater physical activity (36, 37). Furthermore, alcohol consumption and being an only child are associated with lower levels of health-promoting behaviors, which aligns with previous research (38). In this study, a deeper analysis of demographic factors reveals that female students, those with less-educated parents, non-only children, and students without a history of smoking or drinking tend to have higher levels of health-promoting behaviors. This may be because females are more concerned with health, and students from these backgrounds may be more inclined to enhance their health through daily lifestyle choices, taking responsibility, and engaging in social interactions (39). For college students, reduced smartphone dependence can increase the likelihood of engaging in health-promoting behaviors during leisure time, thus enhancing overall health. However, smartphone dependence shortens their available time for such activities (20, 40). Moreover, when students shift their focus toward health-promoting behaviors, the impact of smartphone dependence on sleep quality diminishes. These findings are significant for preventing smartphone dependence and improving sleep quality among college students.

The analysis of the combined mediation effects of negative emotions and health-promoting behaviors reveals that smartphone dependence not only directly influences negative emotions among college students but also indirectly impacts sleep quality through the mediation of negative emotions and health-promoting behaviors. Negative emotions, particularly depression, anxiety, and stress, significantly negatively affect health-promoting behaviors. The accumulation of negative emotions exacerbates the impact of smartphone dependence on sleep disturbances, while improving health-promoting behaviors can reduce smartphone dependence and enhance sleep quality. Specifically, increased smartphone dependence leads to greater accumulation of negative emotions, which in turn reduces physical activity, interpersonal interactions, and healthy dietary choices among students. This creates a cycle where reduced health-promoting behaviors fail to alleviate negative emotions, leading to worsened sleep quality and more severe sleep disorders. Previous studies have shown a bidirectional predictive relationship between negative emotions and health-promoting behaviors, where improving health behaviors can help alleviate negative emotions and vice versa (17, 41). This study confirms that the accumulation of negative emotions indeed leads to a decline in health-promoting behaviors, intensifying the impact of smartphone dependence on sleep disorders. On the other hand, enhancing health-promoting behaviors helps mitigate negative emotions, reduce smartphone dependence, and improve sleep quality. It is worth noting that while exploring the effects of smartphone addiction on sleep quality and mental health, the potential benefits of smartphone use, such as its role in improving negative emotions and sleep quality through apps and AI technologies, should not be overlooked. However, this study does not delve into these aspects. Therefore, promoting health-promoting behaviors is crucial for alleviating negative emotions, reducing smartphone dependence, and improving sleep quality among college students.

### 4.1 Limitations and future directions

Although this study, through the chain mediation effects of negative emotions and health-promoting behaviors, in conjunction with previous research, identified the direct impact of smartphone dependence on sleep quality, as well as its indirect effects via these mediators, several limitations remain. First, the study’s cross-sectional design only allows for speculative conclusions about the bidirectional relationships between smartphone dependence, negative emotions, and health-promoting behaviors based on existing literature. Future research should address this by conducting longitudinal studies with larger sample sizes, using cross-lagged or longitudinal mediation models to establish causal relationships. Second, while demographic factors were considered as covariates, they were not examined as predictors of the mediation effects. Future research should investigate how different demographic factors influence these mediation pathways to provide a more comprehensive understanding. Third, the dual nature of smartphones, as both beneficial and harmful, was not fully explored. While the study focused on the detrimental effects of smartphone dependence on negative emotions, health-promoting behaviors, and sleep quality, it overlooked potential positive impacts, such as AI-assisted interventions for mental health and sleep. This area warrants further exploration. Finally, the study only considered negative emotions and health-promoting behaviors as mediators of the relationship between smartphone dependence and sleep quality. Given the complexity and diversity of college life, future research should explore additional factors and develop more comprehensive models to better understand this relationship.

## 5 Conclusion

Smartphone dependence and negative emotions both positively influence sleep quality among college students, while health-promoting behaviors have a negative impact on it. Smartphone dependence directly affects sleep quality and also exerts indirect effects through the mediating roles of negative emotions and health-promoting behaviors, both independently and in a chain. Different demographic factors (e.g., gender, residence) lead to variations in these relationships, suggesting that future research should employ more detailed subgroup analyses to better understand the potential influences on sleep quality among college students.

## Data Availability

The original contributions presented in the study are included in the article/supplementary material, further inquiries can be directed to the corresponding author.

## References

[1] Chinese Internet Data Research and Information Center-199IT. CNNIC: The 53rd Statistical Report on the Development of China’s Internet Internet Data Information Network-199IT. (2024) Available at: https://www.199it.com/archives/1682273.html#google_vignette (Accessed June 19, 2024).

[2] Chinese Sleep Research Society. (2024) Available at: http://www.zgsmyjh.org (Accessed June 22, 2024).

[3] Long J Liu T-Q Liao Y-H Qi C He H-Y Chen S-B. Prevalence and correlates of problematic smartphone use in a large random sample of Chinese undergraduates. BMC Psychiatr. (2016) 16:408. doi: 10.1186/s12888-016-1083-3, PMID: 27855666 PMC5114822

[4] Li Y Li G Liu L Wu H. Correlations between mobile phone addiction and anxiety, depression, impulsivity, and poor sleep quality among college students: a systematic review and meta-analysis. J Behav Addict. (2020) 9:551–71. doi: 10.1556/2006.2020.00057, PMID: 32903205 PMC8943681

[5] Bianchi A Phillips JG. Psychological predictors of problem mobile phone use. Cyberpsychol Behav Impact Int Multimed Virtual Real Behav Soc. (2005) 8:39–51. doi: 10.1089/cpb.2005.8.39, PMID: 15738692

[6] Gao W-J Hu Y Ji J-L Liu X-Q. Relationship between depression, smartphone addiction, and sleep among Chinese engineering students during the COVID-19 pandemic. World J Psychiatr. (2023) 13:361–75. doi: 10.5498/wjp.v13.i6.361, PMID: 37383286 PMC10294134

[7] Carter B Rees P Hale L Bhattacharjee D Paradkar MS. Association between portable screen-based media device access or use and sleep outcomes: a systematic review and Meta-analysis. JAMA Pediatr. (2016) 170:1202–8. doi: 10.1001/jamapediatrics.2016.2341, PMID: 27802500 PMC5380441

[8] Yang L-L Guo C Li G-Y Gan K-P Luo J-H. Mobile phone addiction and mental health: the roles of sleep quality and perceived social support. Front Psychol. (2023) 14:1265400. doi: 10.3389/fpsyg.2023.1265400, PMID: 37809316 PMC10556235

[9] Precht L-M Mertens F Brickau DS Kramm RJ Margraf J Stirnberg J. Engaging in physical activity instead of (over)using the smartphone: an experimental investigation of lifestyle interventions to prevent problematic smartphone use and to promote mental health. Z Gesundheitswissenschaften J Public Health. (2023) 32:589–607. doi: 10.1007/s10389-023-01832-5, PMID: 36785655 PMC9909154

[10] Cui G Yin Y Li S Chen L Liu X Tang K. Longitudinal relationships among problematic mobile phone use, bedtime procrastination, sleep quality and depressive symptoms in Chinese college students: a cross-lagged panel analysis. BMC Psychiatry. (2021) 21:449. doi: 10.1186/s12888-021-03451-4, PMID: 34507561 PMC8431882

[11] Liu M Lu C. Mobile phone addiction and depressive symptoms among Chinese university students: the mediating role of sleep disturbances and the moderating role of gender. Front Public Health. (2022) 10:965135. doi: 10.3389/fpubh.2022.965135, PMID: 36203678 PMC9531624

[12] Jin Y Wang Y Liu S Niu S Sun H Liu Y. The relationship between stressful life events and depressive symptoms in college students: mediation by parenting style and Gender’s moderating effect. Psychol Res Behav Manag. (2024) 17:1975–89. doi: 10.2147/PRBM.S461164, PMID: 38766317 PMC11100962

[13] Liu A Shi Y Zhao Y Ni J. Influence of academic involution atmosphere on college students’ stress response: the chain mediating effect of relative deprivation and academic involution. BMC Public Health. (2024) 24:870. doi: 10.1186/s12889-024-18347-7, PMID: 38515074 PMC10956225

[14] Liu X Zhang L Zhang X. Sleep quality and emotional adaptation among freshmen in elite Chinese universities during prolonged COVID-19 lockdown: the mediating role of anxiety symptoms. Int J Ment Health Promot. (2024) 26:105–16. doi: 10.32604/ijmhp.2023.042359

[15] Liu Q Yang X Zhang C Xiong J. Is decreasing problematic mobile phone use a pathway for alleviating adolescent depression and sleep disorders? A randomized controlled trial testing the effectiveness of an eight-session mindfulness-based intervention. J Behav Addict. (2024) 13:525–41. doi: 10.1556/2006.2024.00034, PMID: 38905005 PMC11220812

[16] Liu H Liu Y Li B. Predictive analysis of health/physical fitness in health-promoting lifestyle of adolescents. Front Public Health. (2021) 9:691669. doi: 10.3389/fpubh.2021.691669 34490182 PMC8416607

[17] Tao Y Wu J Huang L Zheng K Liu H Tian H. The relationship between health-promoting behaviors and negative emotions in college freshmen: a cross-lagged analysis. Front Public Health. (2024) 12:1348416. doi: 10.3389/fpubh.2024.1348416 38737866 PMC11088242

[18] Riera-Sampol A Rodas L Martínez S Moir HJ Tauler P. Caffeine intake among undergraduate students: sex differences, sources, motivations, and associations with smoking status and self-reported sleep quality. Nutrients. (2022) 14:1661. doi: 10.3390/nu14081661, PMID: 35458223 PMC9029267

[19] Ge Y Xin S Luan D Zou Z Liu M Bai X. Association of physical activity, sedentary time, and sleep duration on the health-related quality of life of college students in Northeast China. Health Qual Life Outcomes. (2019) 17:124. doi: 10.1186/s12955-019-1194-x, PMID: 31311564 PMC6636029

[20] Wang P-Y Chen K-L Yang S-Y Lin P-H. Relationship of sleep quality, smartphone dependence, and health-related behaviors in female junior college students. PLoS One. (2019) 14:e0214769. doi: 10.1371/journal.pone.0214769, PMID: 30943270 PMC6447181

[21] Walsh SP White KM Young RM. Needing to connect: the effect of self and others on young people’s involvement with their mobile phones. Aust J Psychol. (2010) 62:194–203. doi: 10.1080/00049530903567229

[22] Kang Y Liu S Yang L Xu B Lin L Xie L. Testing the bidirectional associations of Mobile phone addiction behaviors with mental distress, sleep disturbances, and sleep patterns: a one-year prospective study among Chinese college students. Front Psych. (2020) 11:634. doi: 10.3389/fpsyt.2020.00634, PMID: 32765310 PMC7379372

[23] Lin L Xu X Fang L Xie L Ling X Chen Y. Validity and reliability of the Chinese version of Mobile phone involvement questionnaire in college students. Nan Fang Yi Ke Da Xue Xue Bao. (2020) 40:746–51. doi: 10.12122/j.issn.1673-4254.2020.05.22, PMID: 32897215 PMC7277304

[24] Buysse DJ Reynolds CF Monk TH Berman SR Kupfer DJ. The Pittsburgh sleep quality index: a new instrument for psychiatric practice and research. Psychiatry Res. (1989) 28:193–213. doi: 10.1016/0165-1781(89)90047-4, PMID: 2748771

[25] Tsai P-S Wang S-Y Wang M-Y Su C-T Yang T-T Huang C-J. Psychometric evaluation of the Chinese version of the Pittsburgh sleep quality index (CPSQI) in primary insomnia and control subjects. Qual Life Res. (2005) 14:1943–52. doi: 10.1007/s11136-005-4346-x, PMID: 16155782

[26] Lovibond SH Lovibond PF. Depression anxiety stress scales. Psychol Assessment. (2011). doi: 10.1037/t01004-000

[27] Chan RCK Xu T Huang J Wang Y Zhao Q Shum DHK. Extending the utility of the depression anxiety stress scale by examining its psychometric properties in Chinese settings. Psychiatry Res. (2012) 200:879–83. doi: 10.1016/j.psychres.2012.06.041, PMID: 22921506

[28] Walker SN Sechrist KR Pender NJ. Health promotion model-instruments to measure health promoting lifestyle: Health-promoting lifestyle profile [HPLP II] (adult version). (1995) Available at: http://deepblue.lib.umich.edu/handle/2027.42/85349 (Accessed August 13, 2024).

[29] Zhao Z Kou Y. Effect of short video addiction on the sleep quality of college students: chain intermediary effects of physical activity and procrastination behavior. Front Psychol. (2024) 14:1287735. doi: 10.3389/fpsyg.2023.1287735 38274685 PMC10808398

[30] Kaya F Bostanci Daştan N Durar E. Smart phone usage, sleep quality and depression in university students. Int J Soc Psychiatry. (2021) 67:407–14. doi: 10.1177/0020764020960207, PMID: 32969293

[31] Lissak G. Adverse physiological and psychological effects of screen time on children and adolescents: literature review and case study. Environ Res. (2018) 164:149–57. doi: 10.1016/j.envres.2018.01.015, PMID: 29499467

[32] Krizan Z Boehm NA Strauel CB. How emotions impact sleep: a quantitative review of experiments. Sleep Med Rev. (2024) 74:101890. doi: 10.1016/j.smrv.2023.101890, PMID: 38154235

[33] Zhu Y Meng R Jiang C Yang N Huang M Wang X. Sleep quality and subjective well-being in healthcare students: examining the role of anxiety and depression. Front Public Health. (2023) 11:1281571. doi: 10.3389/fpubh.2023.1281571, PMID: 38213643 PMC10784115

[34] Wang F Bíró É. Determinants of sleep quality in college students: a literature review. Explore N Y N. (2021) 17:170–7. doi: 10.1016/j.explore.2020.11.003 33246805

[35] Ying Z-Q Li D-L Liang G Yin Z-J Li Y-Z Ma R. Reduced health-related quality of life due to Mobile phone dependence in a sample of Chinese college students: the mediating role of chronotype and sleep quality. Am J Health Promot AJHP. (2024) 12:8901171241258375. doi: 10.1177/08901171241258375, PMID: 38831423

[36] Wei C-N Harada K Ueda K Fukumoto K Minamoto K Ueda A. Assessment of health-promoting lifestyle profile in Japanese university students. Environ Health Prev Med. (2012) 17:222–7. doi: 10.1007/s12199-011-0244-8, PMID: 21987366 PMC3348247

[37] Lee RLT Loke AJTY. Health-promoting behaviors and psychosocial well-being of university students in Hong Kong. Public Health Nurs. (2005) 22:209–20. doi: 10.1111/j.0737-1209.2005.220304.x 15982194

[38] Kazemi DM Levine MJ Dmochowski J Roger Van Horn K Qi L. Health behaviors of mandated and voluntary students in a motivational intervention program. Prev Med Rep. (2015) 2:423–8. doi: 10.1016/j.pmedr.2015.05.004, PMID: 26844100 PMC4721403

[39] Fakhrunnisak D Patria B. The positive effects of parents’ education level on children’s mental health in Indonesia: a result of longitudinal survey. BMC Public Health. (2022) 22:949. doi: 10.1186/s12889-022-13380-w, PMID: 35549703 PMC9097111

[40] Zhu W Liu J Lou H Mu F Li B. Influence of smartphone addiction on sleep quality of college students: the regulatory effect of physical exercise behavior. PLoS One. (2024) 19:e0307162. doi: 10.1371/journal.pone.0307162, PMID: 39058670 PMC11280214

[41] Wang Y Ma Q. The impact of social isolation on smartphone addiction among college students: the multiple mediating effects of loneliness and COVID-19 anxiety. Front Psychol. (2024) 15:1391415. doi: 10.3389/fpsyg.2024.1391415 39105145 PMC11299513

